# Identification of Long Noncoding RNAs as Predictors of Survival in Triple-Negative Breast Cancer Based on Network Analysis

**DOI:** 10.1155/2020/8970340

**Published:** 2020-03-03

**Authors:** Xiao-Xiao Li, Li-Juan Wang, Jie Hou, Hong-Yang Liu, Rui Wang, Chao Wang, Wen-Hai Xie

**Affiliations:** ^1^School of Life Sciences, Shandong University of Technology, Zibo, China; ^2^Institute of Biomedical Research, Shandong University of Technology, Shandong Provincial Research Center for Bioinformatics Engineering and Technique, Zibo Key Laboratory of New Drug Development of Neurodegenerative Diseases, Zibo, China

## Abstract

Breast cancer is the most common cancer observed in adult females, worldwide. Due to the heterogeneity and varied molecular subtypes of breast cancer, the molecular mechanisms underlying carcinogenesis in different subtypes of breast cancer are distinct. Recently, long noncoding RNAs (lncRNAs) have been shown to be oncogenic or play important roles in cancer suppression and are used as biomarkers for diagnosis and therapy. In this study, we identified 134 lncRNAs and 6,414 coding genes were differentially expressed in triple-negative (TN), human epidermal growth factor receptor 2- (HER2-) positive, luminal A-positive, and luminal B-positive breast cancer. Of these, 37 lncRNAs were found to be dysregulated in all four subtypes of breast cancers. Subtypes of breast cancer special modules and lncRNA-mRNA interaction networks were constructed through weighted gene coexpression network analysis (WGCNA). Survival analysis of another public datasets was used to verify the identified lncRNAs exhibiting potential indicative roles in TN prognosis. Results from heat map analysis of the identified lncRNAs revealed that five blocks were significantly displayed. High expressions of lncRNAs, including LINC00911, CSMD2-AS1, LINC01192, SNHG19, DSCAM-AS1, PCAT4, ACVR28-AS1, and CNTFR-AS1, and low expressions of THAP9-AS1, MALAT1, TUG1, CAHM, FAM2011, NNT-AS1, COX10-AS1, and RPARP-AS1 were associated with low survival possibility in TN breast cancers. This study provides novel lncRNAs as potential biomarkers for the therapeutic and prognostic classification of different breast cancer subtypes.

## 1. Introduction

Breast cancer is the most frequently occurring cancer and the second cause of female cancer mortality worldwide. According to a report from the International Agency for Research on Cancer, breast cancer accounts for approximately 11.6% of cancer incidences [1]. Although deaths caused by breast cancer have declined over time due to the development of new diagnostic and therapeutic strategies, the molecular mechanisms underlying this disease remain to be revealed. As breast cancers are notoriously heterogeneous, clinical and morphologic signatures are diversified. Consequently, the exact classification of breast cancer is a challenge for clinicians and scientist. Variations in the expressions of the estrogen receptor (ER), human epidermal growth factor receptor 2 (HER2), and progesterone receptor (PR) can be used to classify breast cancers into four major intrinsic molecular subtypes: triple-negative (TN), luminal A, luminal B, and HER2-positive [2]. TN breast cancers are well-known as lacking ER and PR as well as HER2 [3]. Luminal A breast cancers are defined as ER-positive, HER2-negative, and “low” recurrence risk, partially expressing PR [4]. Luminal B breast cancers are characterized by HER2-positive, ER and PR-negative, and a high histologic grade [5]. HER2-positive breast cancers are defined as HER2-positive and lacking of ER and PR. Along with ER, PR, and HER2, many other molecular genes also have been used to subtype breast cancers. High expression of Ki67, known as a proliferation marker, was usually been used to define breast cancer subtypes [6]. Molecular classifications are using multigene assays to classify breast cancers into low- and high-risk groups for personalized therapy [7, 8]. Many genes have been found as signatures in the diagnosis and prognosis of breast cancer. Decreased levels of uPA and PAI-1 are associated with lower risk of cancer recurrence, and overexpression of Ki67, cyclin D, cyclin E, p27, and p21 indicates uncontrolled tumor cell proliferation [9]. The molecular interaction regulation mechanisms mediating heterogeneity and different molecular subtypes of breast cancer need to be further probed.

Long noncoding RNAs (lncRNAs), which are 200 nucleotides in length and play important roles in various biological pathways, have been shown to be oncogenic or play important roles in cancer suppression and are used as biomarkers for diagnosis and therapy [10, 11]. Aberrant expressions of lncRNAs, such as MALAT1 [12], DANCR [13], PDCD4-AS1 and [14], MIR100HG [15], are closely associated with the occurrence and progression of breast cancer. Overexpression of HOTAIR stimulates the invasion and metastasis of breast cancer cells [16]. PIWI-interacting noncoding RNA especially expressed in breast cancer is a response to estrogen regulation in breast cancer cells [17]. lncRNAs can also mediate the progression of breast cancer by interacting with microRNAs. For example, MEG3 acts as a suppressor to inhibit cell epithelial-mesenchymal transition by sponging miR-421 and targeting E-cadherin in breast cancer [18]. Highly expressed H19/low miR-675 and low NEAT1/high miR-204 could differentiate breast cancer subtypes and could be considered as diagnostic and therapeutic biomarkers [19]. lncRNAs can also act as epigenetic regulators affecting histone modifications and stabilize signal complexes and nuclear structures by recruiting chromatin modification factors [20]. Although a growing volume of lncRNAs has been found playing varied roles in the pathogenesis of breast cancer and drug resistance, their biological participation mechanisms are not yet fully understood.

**Figure 1:**
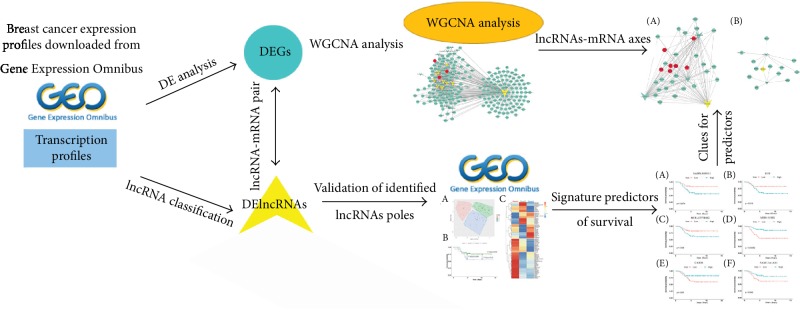
Flow diagram of this study. First, breast cancer expression profiles were downloaded from Gene Expression Omnibus. Second, the pipeline of lncRNA classification was utilized to identify lncRNA expression. Third, constructed and analysis breast cancers associated network-based on WGCNA. Finally, validation of the prediction roles of identified lncRNAs in TN breast cancers was performed by survival analysis.

TN breast cancer is a heterogeneous subtype of breast cancers with poor prognosis and is beginning to be refined by molecular characteristics. Based on gene expression profiles, six TN breast cancer subtypes were classified [21]. As the high heterogeneous of TN breast cancer, it is necessary to disclose its molecular characteristics and identify effective clinical strategy to improve the therapeutic approach. A series of lncRNAs has been found dysregulated in TN breast cancer [22]. lncRNAs are involved in the regulation of most mammalian protein coding genes, which are responsible for various cellular processes such as cell differentiation, development, proliferation, and apoptosis. Exploitation of the function of dysregulated lncRNAs will provide potential clinical applications for TN's diagnosis and treatment. WGCNA can be used to identify key lncRNAs associated with multiple cancer pathogenesis and progression [23–26]. Survival analysis verified the identified lncRNAs possessing potential indicative roles in TN prognosis. These survival expectation indictors associated with coexpressing coding genes formed coexpressed axes, which could act as attractive therapeutic targets for the treatment of TN breast cancer. The workflow of this study is summarized in Figure 1.

## 2. Materials and Methods

### 2.1. Breast Cancer Expression Profiles Downloaded from Gene Expression Omnibus

The public datasets GSE45827 and GSE58812 were downloaded from the NCBI Gene Expression Omnibus (GEO). GSE45827 includes 41 TN, 30 HER2, 29 luminal A, and 30 luminal B breast cancer patients and 11 normal tissue samples [27]. GSE58812 includes 107 triple-negative breast cancer patients [28]. Normal tissue samples were used as a control to identify differentially expressed coding genes (DEGs) and differentially expressed lncRNAs (DElncRNAs). In order to reduce the bias of different data resources, datasets adopted in this study were both based on the same GPL570 platform. The type of microarray was Affymetrix Human Genome U133 plus 2.0 Array. All raw data preprocessing was conducted in the R software environment. The Affy package was adopted to process the initial datasets [29]. A robust multiarray average function was used to detect and normalize the expression of probes. The Limma package was used for differential expression analysis [30]. The Benjamini and Hochberg method was used to control the false discovery rate (FDR). The adjusted *p* values were used to control the FDR. The FDR ratio was set as 5%. Adjusted *p* values < 0.05 and log (fold change) > 1 were selected as the thresholds for DEGs and DElncRNAs.

### 2.2. lncRNA Classification Pipeline

The pipeline of lncRNA classification was utilized to identify lncRNA expression as previously described [31], with minor modifications. The Affymetrix HG-U133 plus 2.0 probe set ID was mapped to the latest NetAffx annotation files. First, we extracted the probe sets with RefSeq IDs labelled as “NR_”. For the selected probe sets annotated with Ensembl gene IDs, we annotated with lncRNA, processed the transcript noncoding and antisense according to Homo_sapiens.GRCH38.96.gtf. The last probe sets without Ensembl gene IDs were filtered using pseudogenes, rRNAs, microRNAs, snoRNA, and tRNAs according to NetAffx annotation files. Finally, we obtained 2,553 Affymetrix probe IDs, which were annotated as lncRNA transcripts (1,870 annotated lncRNAs).

### 2.3. Construction of a Weighted Gene Coexpression Network and Identification of Modules Associated with Different Subtypes of Breast Cancer

WGCNA was adopted to identify coexpression modules [32]. The signed weighted correlation network was constructed by the first creating a matrix of pairwise correlations between all pairs of genes [33]. The power of seven was interpreted as a soft threshold of the correlation matrix. Depending on the resulting adjacency matrix, we calculated the topological overlap matrix, which measures the interconnectedness of the coexpression network. Then, genes with highly similar coexpression relationships were grouped together by hierarchical clustering based on the topological overlap matrix. The Dynamic Hybrid Tree Cut algorithm was adopted to define modules, meaning the coexpression genes. Module eigengenes were defined as the first principal component to represent each module. Finally, modules were merged based on correlation above 0.85. After identifying the coexpression modules, we associated modules with each sample type. Modules with correlation > 0.5 (*p* < 0.01) were picked up as respective subtypes of breast cancer.

### 2.4. Construction and Analysis of lncRNA-mRNA Coexpression Network

Based on the Pearson correlation coefficient of lncRNA-mRNA, we constructed breast cancer-related lncRNA-mRNA coexpression networks. The threshold of selection for lncRNA-mRNA pairs was set at an absolute value of the Pearson correlation coefficient above 0.6. Topological analysis of the coexpression network was performed using the Cytoscape (version 3.7.1) software [34]. Hub genes were determined using high connectivity of genes by summing the connection strengths with other module genes.

### 2.5. Gene Ontology Analysis

Gene ontology analysis of identified modules and core genes was performed using DAVID 6.8 [35]. The corrected FDR of a *p* value < 0.05 was selected as the threshold for enrichment of the GO_FAT terms.

### 2.6. Validation of Identified lncRNA Roles in TN Cancer Classification

The dataset GSE58812 was used to verify the important roles of identified DElncRNAs. Preprocessing of GSE58812 was conducted in the R software as described above. After obtaining the expression matrix of the datasets, these TN cancer samples were classified into groups based on principal component analysis. The clustering was performed by package ggfortify of R [36]. As the raw reference of GSE58812 classified the 107 TN breast cancer samples into three subtypes, we set the cluster number as 3. According to the clinical data of these TN cancer samples, survival analysis was performed using survminer package [37]. The median of DElncRNA expressions among different TN cancer sample groups was adopted to display the panel of predicting survival expectation.

## 3. Results

### 3.1. DEGs and DElncRNAs in Different Subtypes of Breast Cancer Patients

**Figure 2:**
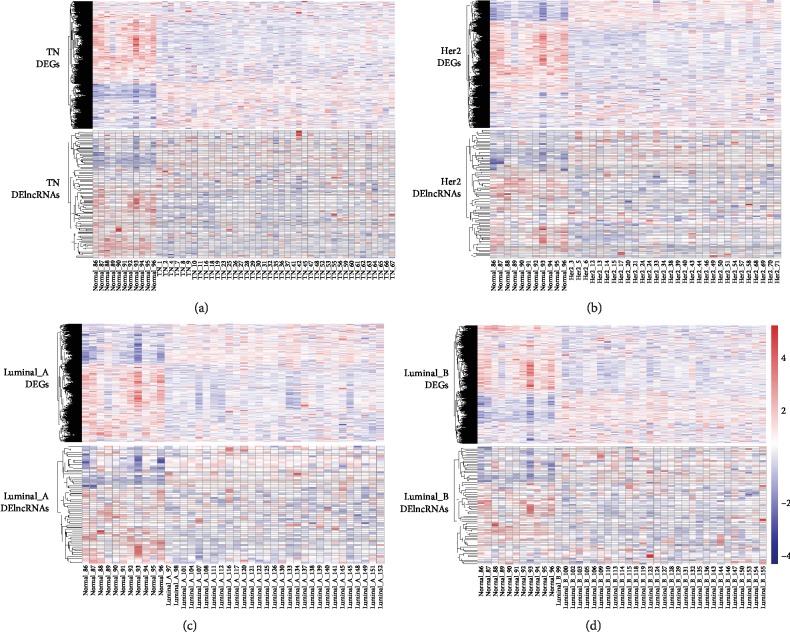
Identification of differentially expressed coding genes and lncRNAs among the four molecular subtypes of breast cancers. Hierarchical analysis showed differentially expressed coding genes and lncRNAs compared to normal tissue samples. The upper chart was for DEGs and the bottom for DElncRNAs (a–d). TN breast cancers identified 3,514 DEGs and 96 DElncRNAs (a). HER2 breast cancers identified 3,181 DEGs and 87 DElncRNAs (b). Luminal A breast cancers identified 2,198 DEGs and 60 DElncRNAs (c). Luminal B breast cancers identified 2,841 DEGs and 70 DElncRNAs (d).

**Figure 3:**
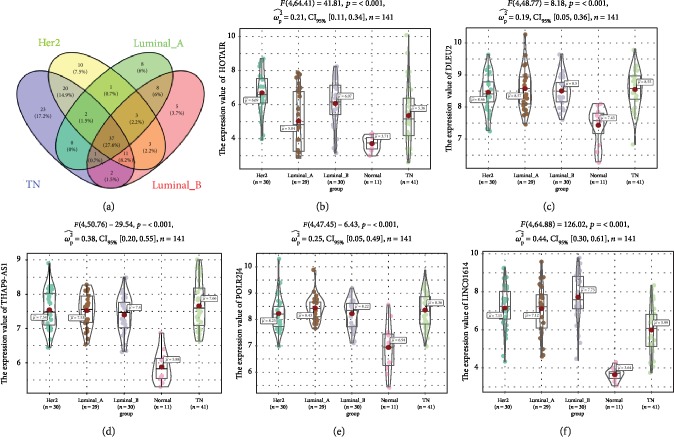
Venn diagram of DElncRNAs among the four molecular subtypes of breast cancer. Venn diagram shows the interactions of DElncRNAs among the four different molecular subtypes of breast cancer (a). The expression of five upregulated DElncRNAs in the four different subtypes of breast cancer and normal tissues was plotted by ggstatsplot (b–f). The expression values of upregulated DElncRNAs were shown as HOTAIR (b), DLEU2 (c), THAP9-AS1 (d), POLR2J4 (e), and LINC01614 (f).

After applying lncRNA classification, we annotated the microarray with 17,242 coding genes and 1,870 lncRNAs. We identified 3,514 coding genes and 96 lncRNAs, 3,181 coding genes and 87 lncRNAs, 2,198 coding genes and 60 lncRNAs, and 2,841 coding genes and 70 lncRNAs differentially expressed in TN, HER2, luminal A, and luminal B breast cancers compared to normal tissues (Figure 2). The four different subtypes of breast cancer shared 37 common differentially expressed lncRNAs (Figure 3(a)). Most of the 37 common differentially expressed noncoding RNAs were downregulated in the four different subtypes of breast cancers compared to normal tissues (Table 1). This means that the downregulated lncRNAs may be the tumor suppressor genes in the normal tissues. Only five lncRNAs (HOTAIR, DLEU2, THAP9-AS1, POLR2J4, and LINC01614) were upregulated (Figures 3(e)–3(f)). LncRNA HOTAIR has been proven to play a significant role in promoting invasion of breast cancer cells [38]. DLEU2 was reported to play a role in response to estrogen regulation in human breast cancer cell line [39]. POLR2J4 has also been used to predict recurrence-free survival in hepatocellular carcinoma [40]. Knockdown of LINC01614 inhibits lung adenocarcinoma cell progression [41]. THAP9-AS1 was the first time observed to be associated with breast cancer.

### 3.2. WGCNA Analysis of the Coding Genes and lncRNAs

**Figure 4:**
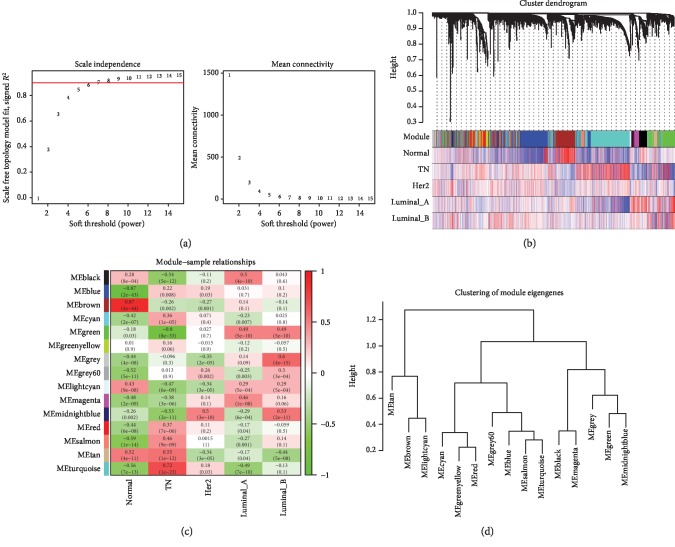
Fifteen gene modules were identified based on differentially expressed coding genes and lncRNAs. Selection of soft thresholding powers for WGCNA (a). Red line corresponds to 0.9. As the lowest power satisfies the approximate scale-free topology criterion, number 7 was interpreted as a soft threshold of the correlation matrix (a). Heat map of gene dendrogram assigned to the four different subtypes of breast cancer. The color row was painted according to expression values of genes in the dendrogram (b). Relationships of module eigengenes and different breast cancer subtypes showed in these datasets. Each row in the table corresponds to the identified 15 gene modules, and each column corresponds to the sample types. The correlations of the corresponding module eigengenes, samples, and *p* values were labelled in the table. The colors of the tables were painted corresponding to the correlation between gene modules and samples (c). 15 gene module eigengenes were classified into three groups by hierarchical clustering (d).

The combination of these differentially expressed coding genes and lncRNAs formed a matrix dataset including 6,414 genes and 134 lncRNAs among 141 samples. The network was constructed with the soft threshold power of 7 (Figure 4(a)). We identified 15 gene modules based on a minimum module size of 30 merged modules with eigengene correlation above 0.85. Modules related to different breast cancer subtypes are shown in Figure 4(b). A gene dendrogram was obtained using average linkage hierarchical clustering. The color row was painted according to the expression values of genes in the dendrogram. The turquoise module was highly associated with TN, and midnight blue was related to HER2 and luminal B breast cancers. Luminal B breast cancer was highly associated with the grey modules compared to other subtypes of breast cancers. Luminal A has three relatively high modules including black, green, and magenta. Luminal A and luminal B have the same relationship with the green modules (Figure 4(c)). These related modules will provide more information for subtyping of different breast cancers. In addition, the brown and blue modules were, respectively, positively and negatively correlated with normal tissue samples. The 15 gene modules eigengenes were classified into three groups using hierarchical clustering (Figure 4(d)).

### 3.3. Construction of Breast Cancer-Related lncRNA-mRNA Coexpression Networks

**Figure 5:**
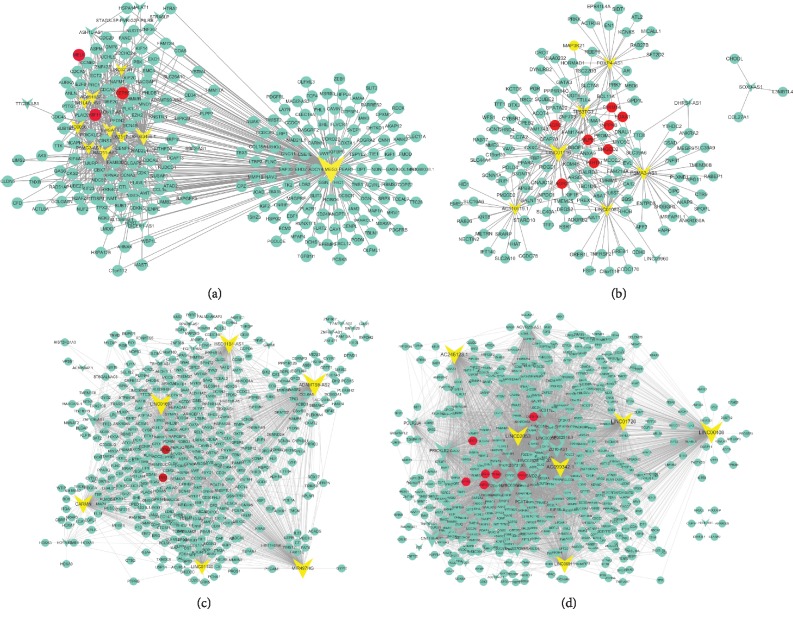
Results of different breast cancer subtype-related lncRNA-mRNA coexpression networks. The coexpression networks of TN-related module—turquoise (a), luminal A- and luminal B-related module—green (b), normal-related module—brown (c), and normal negative-related module—blue (d). Nodes with a V shape arrow are lncRNAs. Nodes with ellipse are mRNAs. Key lncRNAs and mRNAs in the network are, respectively, highlighted in yellow and red colors. The size of the nodes corresponds to their degrees.

Based on the Pearson correlation coefficients of lncRNA-mRNA, we constructed breast cancer-related lncRNA-mRNA coexpression networks using WGCNA. The threshold for the selection of lncRNA-mRNA pairs was set at an absolute value of the Pearson correlation coefficient above 0.6. The TN-related lncRNA-mRNA coexpression network derived from the turquoise module consists of 19 lncRNAs and 218 DEGs (Figure 5(a)). Maternally expressed gene 3 (MEG3) was the hub of lncRNA in the constructed network with 219 interaction links. Previous studies showed that MEG3 was downregulated in various types of cancers including breast cancer. Meta-analysis demonstrated that low expression of MEG3 is associated with poor survival in cancer patients [42]. GO analysis of MEG3 linked 219 genes, which enriched cell adhesion, cell proliferation, immune system processes, and other processes. The network could provide useful information for the mechanism of MEG3. CEP55, MELK, and KIF11 were the top 3 degree DEGs in the network. CEP55 is a determinant of cell fate during perturbed mitosis in breast cancer [43].

Luminal A- and luminal B-related lncRNA-mRNA coexpression network derived from the green module consists of 9 lncRNAs and 144 DEGs. LINC01116, LINC01087, AC110619, FOXP4-AS1, and TP53TG1 were the top nodes in this network, while MLPH, AGR2, SMIM4, FOXA1, INPP4B, MAGED, P4HTM, and AGR3 mediated the connection of the whole network.

Normal tissue-related lncRNA-mRNA coexpression network derived from the brown module consists of 23 lncRNAs and 405 DEGs. The top nodes in this network were ADMTS9-AS2, HSD11B1-AS1, LINC01697, MIR497HG, LINC01140, and CARMN. BTNL9 and TNS1 were the top degree DEGs in the constructed network. TNS1 modulated the activation of Cdc42 to regulate cell invasion in breast cancer [44]. The nonnormal-related lncRNA-mRNA coexpression network derived from the blue module was negatively related with normal samples. This cancer-related lncRNA-mRNA coexpression network consists of 38 lncRNAs and 496 DEGs. LINC00408, AC099342.1, LINC02053, LINC01720, and AC245123.1 were the top nodes in this network, while DEFB106A, LECT2, TRIM49, BOLL, OPHN1, ZNF835, and EPPIN were the top degree DEG nodes in the network.

### 3.4. Function Annotation of Breast Cancer-Related lncRNAs

**Figure 6:**
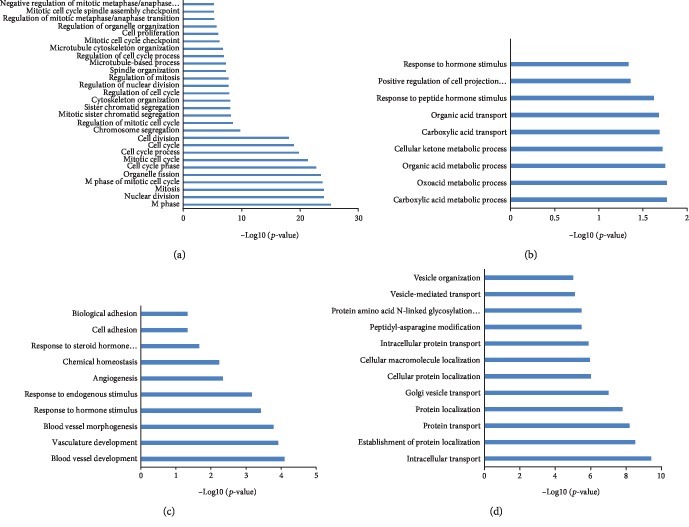
Function annotation of identified DEG-DElncRNA coexpression network. GO analysis shows enriched GO term in TN-related lncRNA-mRNA coexpression networks (a), luminal A- and luminal B-related lncRNA-mRNA coexpression networks (b), normal-related lncRNA-mRNA coexpression network (c), and normal negative-related lncRNA-mRNA coexpression network (d).

We performed function annotation of these constructed lncRNA-mRNA coexpression networks using the DAVID system. The TN-related lncRNA-mRNA coexpression network was involved in cell cycle control and cell proliferation (Figure 6(a)). The current result is consistent with results from previous studies, which showed that overexpression of MEG3 suppresses the proliferation of breast cancer cells [45]. The luminal A- and luminal B-related lncRNA-mRNA coexpression network was involved in carboxylic acid and organic acid transport or metabolic processes and the response to hormone stimulus (Figure 6(b)). That may be the reason why prognosis of these two subtypes of breast cancer is better than that of others. GO analysis of the normal-related lncRNA-mRNA coexpression network enriched blood vessel development and morphogenesis and the angiogenesis term (Figure 6(c)). That means the brown module is essential for normal tissue maintenance. GO analysis of the normal negative-related lncRNA-mRNA coexpression networks enriched protein transport terms such as Golgi vesicle transport and vesicle organization (Figure 6(d)). These lncRNAs interact with coding genes and mediate cancer cell protein transport and cellular morphology during tumorigenesis.

### 3.5. DElncRNAs Act as Survival Expectation Indictors Verified Using Public Datasets

**Figure 7:**
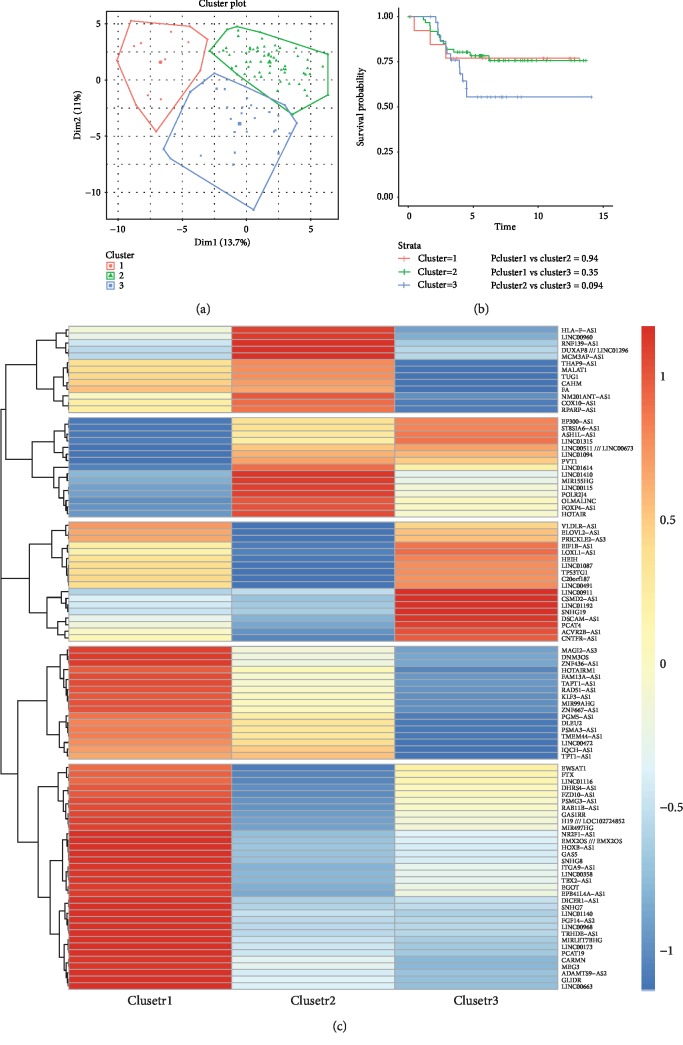
Verification of the inductive roles of identified DElncRNAs based on public datasets. The TN cancer samples were classified into three clusters (a). Survival analysis of the three clusters showed different survival probability (b). The panels of identified 97 lncRNAs act as signature predictors of survival of TN breast cancer patients (c).

The TN breast cancer samples of dataset GSE58812 were classified into three clusters based on the expression of these DElncRNAs (Figure 7(a)). According to the clinical data of these TN cancer samples, survival analysis of the three clusters displayed different survival probabilities. The KM curves with a *p* value of pairwise comparisons for the three clusters are shown in Figure 7(b). The survival probability of cluster 3 was 50% lower than those of cluster 1 or 2 (75%). Five blocks that acted as signature predictors of survival in TN breast cancer patients were significantly displayed using heat map analysis (Figure 7(c)). This means that different DElncRNAs play different roles in TN breast cancer progression by promoting or inhibiting cancer progression. The DElncRNAs THAP9-AS1, MALAT1, TUG1, CAHM, FAM2011, NNT-AS1, COX10-AS1, and RPARP-AS1 were lowly expressed in cluster 3 compared with those of cluster 1 and 2 samples. The analysis of the TCGA database showed that higher expression of TUG1 was associated with better prognosis in breast cancer patients [46]. This is consistent with our bioinformatics prediction. LINC00911, CSMD2-AS1, LINC01192, SNHG19, DSCAM-AS1, PCAT4, ACVR28-AS1, and CNTFR-AS1 were highly expressed in cluster 3. The relative expression values of DElncRNAs can be used as a predictor for survival expectation.

**Figure 8:**
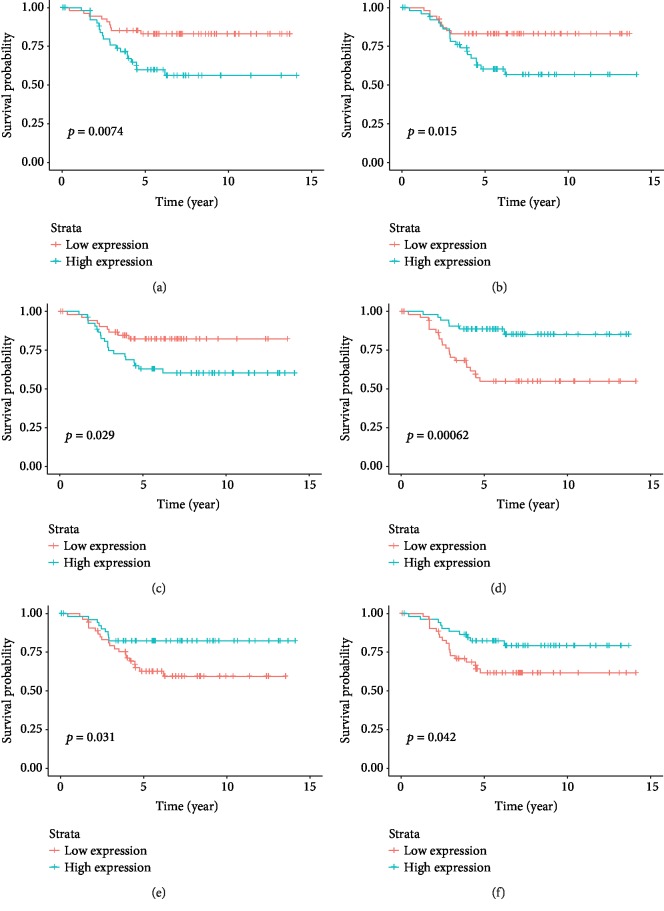
The prediction with significant *p* values between high and low expression values with TN breast cancer prognosis. High expression of LINC00911 (a), H19 (b), and MIRLRT7BHG (c) predicts poor survival in TN breast cancer. High expression of MIR155HG (d), CHAM (e) and FAM13A-AS1 (f), respectively, is associated with higher survival probability.

In order to detect the prediction roles of identified 97 lncRNAs individually, we performed survival analysis of single gene expression and TN breast cancer prognosis. The prediction with significant *p* values between high and low expression values is displayed in Figure 8. High expression of LINC00911 (Figure 8(a)), H19 (Figure 8(b)), and MIRLRT7BHG (Figure 8(c)), respectively, predicted poor survival in TN breast cancer. High expression of MIR155HG (Figure 8(d)), CHAM (Figure 8(e)), and FAM13A-AS1 (Figure 8(f)), respectively, were associated with higher survival probability. This was consistent with the above panel prediction. The individual expression of LINC00911, H19, and MIRLRT7BHG can be used as a predictor for poor prognosis. MIR155HG, CHAM, and FAM13A-AS1 can be used as a positive predictor for survival probability.

### 3.6. The Coexpressing Coding Genes of Survival Expectation Indictors Extracted from the Above lncRNA-mRNA Coexpression Networks

**Figure 9:**
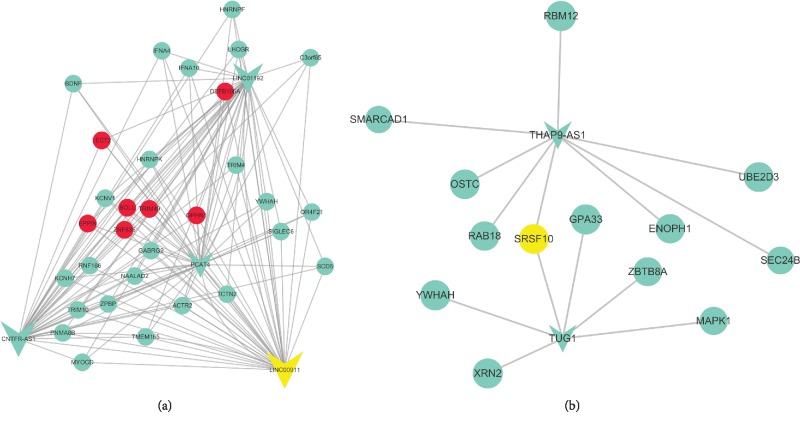
The coexpressing coding genes of survival expectation indictors extracted from the identified lncRNA-mRNA coexpression networks. The four highly expressed lncRNAs associated with 31 coding genes to form a coexpression network (a). Top degree coding genes were painted with red color. The splicing factor SRSF10 bridged those two lowly expressed lncRNAs THAP9-AS1 and TUG1 mediated coexpression network (b). SRSF10 was painted with yellow color.

From the above identified TN, luminal A, luminal B, and normal tissue-associated lncRNA-mRNA coexpression networks, useful interaction information of lncRNAs, and coding genes could be found. LncRNAs LINC00911, LINC01192, PCAT4, and CNTFR-AS1 were listed in the normal samples that were negatively related to the blue module. Extraction of the lncRNA-mRNA interaction network is displayed in Figure 9(a). The four highly expressed lncRNAs associated with 31 coding genes formed a coexpression network. The top degree coding genes were painted with red colors. Several coding genes have been found playing important roles in recurrence, poor diagnosis, and low survival probabilities. Multivariable analyses revealed that LECT2 as one of the predictors for both breast carcinoma recurrence and mortality among smokers [47]. Differentially expressed ZNF835 was associated with ethnicity in colorectal cancer patients [48]. The positive of oligophrenin-1 was significantly correlated with a high Gleason score in prostate cancer [49]. Methyl-DNA binding domain capture technique identified Kcnv1 as a diagnostic marker for early noninvasive detection and subsequent breast cancer surveillance [50]. Brain-derived neurotrophic factor (BDNF) is a potent neurotrophic factor that has been shown to stimulate breast cancer cell growth and metastasis via tyrosine kinase receptors. The methylation of BDNF gene may be a biomarker for suicidal thoughts in patients with breast cancer [51]. BDNF/TrkB pathways activated microRNAs to act as prognostic and predictive biomarkers for detecting patients at a high risk of developing breast cancer [52]. This suggested that lncRNA interacted with Kcnv1 modification methylation levels to mediate survival in TN breast cancer. Increasing expression of SCD5 promoted tumor cell survival in breast cancer [53]. Phosphorylation of hnRNPK S379 participates in the regulation of the migration of triple-negative MDA-MB-231 cells via the EMT signaling pathway [54]. Although there is no information about the function of YWHA, but upregulation of one member of YWHAZ (also known as 14-3-3*ζ*) had been found associated with poor clinical outcome in breast cancer [55].

Cluster 3 lowly expressed lncRNAs, THAP9-AS1 and TUG1, were also listed in the normal samples negatively related to the blue module. Extraction of the lncRNA-mRNA interaction network displayed that the splicing factor SRSF10 bridged those two lowly expressed lncRNAs to mediate the coexpression network (Figure 9(b)). SRSF10, as a splicing factor, has been found playing important roles in colon and cervical cancer oncogenesis by mediating alternative splicing [56, 57]. Whether SRSF10 plays a similar role in breast cancer or not requires more experimental confirmation. The survival expectation indictors associated with coexpressing coding genes formed coexpressed axes, which could act as attractive therapeutic targets for the treatment of TN breast cancer.

## 4. Discussion

Although deaths from breast cancer have declined over time, its molecular mechanism remains to be revealed. Owing to the notorious heterogeneity of breast cancers, it is meaningful to not only classify them based on clinical and morphologic characteristics but also consider intrinsic molecular signatures. Compared with other breast cancers, TN breast cancer was characterized as young onset, high malignancy, easy recurrence, and low survival rates [58]. In addition, TN breast cancer lacks ER and PR as well as HER2 receptor. There are no effective targets of endocrine therapy and targeted therapy for TN breast cancer. It is necessary to identify new molecular targets for TN breast cancer therapy. In our work, we aimed to identify key lncRNAs associated with TN, HER2, luminal A, and luminal B breast cancers. In total, 6,414 coding genes and 134 lncRNAs were found to be dysregulated in different subtypes of breast cancers. A core of 37 lncRNAs was found to be dysregulated in all the four subtypes of breast cancers. Subtypes of breast cancer special modules and lncRNA-mRNA interaction networks were obtained using WGCNA. Moreover, survival analysis of public datasets (GSE58812) verified the identified lncRNAs, which could act as indicators for triple-negative breast cancer prognosis.

Studies on lncRNAs in breast cancer are still in the early stages, and the roles of lncRNAs in breast cancer remain to be elucidated. In the notorious heterogeneity of breast cancers, there is still a core of 37 lncRNAs which dysregulated in all the four major subtypes of breast cancers. Most of the core 37 lncRNAs were downregulated compared to the normal tissues. Only five lncRNAs were upregulated in breast cancer. This indicates that the downregulated lncRNAs may be the tumor suppressor genes in normal tissues. For example, MEG3 is listed in the 32 downregulated core lncRNAs as being downregulated in various types of cancers including breast cancer. Meta-analysis demonstrated that low expression of MEG3 is associated with poor survival in cancer patients [42]. The TN-related lncRNA-mRNA coexpression network derived from the turquoise module displayed MEG3 coexpression pairs. CEP55, MELK, and KIF11 were the top three high degree DEGs in the network. CEP55 is a determinant of cell fate during perturbed mitosis in breast cancer [43]. MELK acts as a potential therapeutic target for TN breast cancer and other aggressive malignancies [59]. A recent study demonstrated that MELK can be upregulated by LINC02418 in colorectal cancer [60]. Recently, a few lncRNAs were reported to function as scaffold molecules recruiting chromatin-modifying complexes to regulate gene expression [61]. lncRNAs can also act as miRNA sponge to reverse miRNA suppression of its target genes [62]. Constructions of breast cancer-related lncRNA-mRNA coexpression networks could provide useful information for lncRNA-mRNA interaction mechanism in breast cancer progression. In this study, we found SRSF10 bridged THAP9-AS1 and TUG1 to mediate the coexpression network. As the splicing factor function of SRSF10, the low expression of THAP9-AS1 and TUG1 in cluster 3 may be related to alternative splicing.

lncRNAs may serve as biomarkers for diagnostic and prognostic purposes and also as potential therapeutic targets in cancer. Several studies have provided insights into the potential clinical implications of lncRNAs in breast cancer. For example, DSCAM-AS1 mediates tumor progression and tamoxifen resistance [63] and HOTAIR reprograms the polycomb repressive complex binding pattern in breast cancer [64]. The relative expression values of DElncRNAs can be used as predictors for survival expectation in TN breast cancer. Interestingly, the expressions of THAP9-AS1 and DLEU2 were relatively lower in cluster 3 and were the only five upregulated lncRNAs in breast cancer samples. There is a paradox between panel lncRNAs and individual lncRNAs prediction of survival probability. H19 and MIRLRT7BHG lined in the last block which means high expression predict high survival probability. High expression of H19 and MIRLRT7BHG was significant related with poor prognosis, since H19 is an estrogen-inducible gene and plays a key role in cell survival and especially in estrogen-induced cell proliferation such as MCF-7 cells [65]. The expression pattern of H19 in TN breast cancers may be different from ER-positive breast cancer. Disclosing the expression pattern of H19 will provide clues for TN breast cancer diagnosis and progression.

Considering the notorious heterogeneity of breast cancers, exact classification needs more molecular markers. In addition, more efforts are needed to detect the relationship between the expressions of these lncRNAs and breast cancer progression. Jiang and colleagues developed a reliable tool to predict tumor recurrence and the benefit of taxane chemotherapy based on an integrated mRNA-lncRNA signature in TN breast cancers [66]. Moreover, further validation in prospective clinical trials of those identified lncRNAs could provide new therapy strategy for the treatment of TN breast cancers. In addition, a huge number of microarray expression profiles of cancer samples are deposited in the public database. With the improvement of annotation information especially, more and more noncoding genes have been found playing important roles in carcinogenesis; mining of those microarray datasets is necessary and would provide novel knowledge about cancer diagnosis and treatment.

We should also point out that there were some limitations in this study. First, the datasets used to identify the differentially expressed DEGs and DElncRNAs were downloaded from a public database and were not validated by quantitation experiments. Second, due to the limit of the sample size, the KM curves with a *p* value of pairwise comparisons for the three TN breast cancer clusters were not statistically different enough. Therefore, further validation of the roles of these lncRNAs would be required in future experiments.

## 5. Conclusions

In conclusion, we identified 134 lncRNAs to be closely related to different subtypes of breast cancers. Based on WGCNA analysis, we constructed breast cancer-related lncRNA-mRNA coexpression networks. The constructed lncRNA-mRNA coexpression networks provided useful information for uncovering the mechanism of lncRNAs in breast cancer genesis. Bioinformatics analysis revealed that lncRNA-mRNA coexpression networks were involved in cell cycle checkpoint, carboxylic acid and organic acid transport, metabolic processes, response to hormone stimulus, Golgi vesicle transport, and vesicle organization. The survival analysis of public datasets verified the identified lncRNAs, which acted as indicators for TN breast cancer prognosis. These survival expectation indictors are associated with coexpressing coding genes to form coexpressed axes, which could act as attractive therapeutic targets for the treatment of TN breast cancer.

## Figures and Tables

**Table 1:** The fold changes of core 37 DElncRNAs in breast cancer compared to normal tissue.

lncRNA symbol	lncRNA Ensembl ID	TN		Her2 positive		Luminal A positive		Luminal B positive	
		Fold change	P.Val	Fold change	P.Val	Fold change	P.Val	Fold change	P.Val
DLEU2^∗^	ENSG00000231607	2.17	2.84*E*‐06	2.04	2.43*E*‐05	2.21	1.32*E*‐05	2.10	2.39*E*‐06
HOTAIR^∗^	ENSG00000228630	3.14	4.22*E*‐03	7.90	5.11*E*‐09	2.51	2.61*E*‐02	5.13	6.74*E*‐06
LINC01614^∗^	ENSG00000230838	5.10	2.32*E*‐07	11.35	3.48*E*‐11	11.18	5.68*E*‐10	16.97	1.30*E*‐11
POLR2J4^∗^	ENSG00000214783	3.44	5.91*E*‐10	3.17	6.65*E*‐10	3.13	7.34*E*‐11	2.87	2.66*E*‐09
THAP9-AS1^∗^	ENSG00000251022	2.68	1.17*E*‐06	2.44	2.61*E*‐05	2.81	1.58*E*‐07	2.43	8.58*E*‐06
AC005064.1	ENSG00000234715	-3.03	2.72*E*‐09	-3.08	1.10*E*‐07	-2.05	1.41*E*‐03	-2.90	2.47*E*‐08
AC099342.1	ENSG00000226097	-4.04	2.82*E*‐14	-4.00	2.79*E*‐11	-3.79	2.66*E*‐10	-3.74	1.84*E*‐10
AC245123.1	ENSG00000277526	-2.21	2.04*E*‐12	-2.18	1.69*E*‐10	-2.28	1.34*E*‐10	-2.23	3.34*E*‐10
ACVR2B-AS1	ENSG00000229589	-3.05	2.04*E*‐15	-3.22	1.69*E*‐13	-2.88	1.17*E*‐10	-3.02	2.71*E*‐12
ADAMTS9-AS2	ENSG00000241684	-5.70	1.03*E*‐16	-5.03	5.99*E*‐13	-3.06	6.45*E*‐07	-5.24	2.51*E*‐14
AP001816.1	ENSG00000254531	-2.42	1.90*E*‐11	-2.10	4.98*E*‐06	-2.09	8.57*E*‐09	-2.05	1.05*E*‐06
AP002518.1	ENSG00000256195	-2.10	4.86*E*‐09	-2.20	8.35*E*‐08	-2.16	1.76*E*‐07	-2.20	1.34*E*‐07
AP003110.1	ENSG00000255084	-2.03	1.19*E*‐07	-2.02	1.98*E*‐06	-2.10	1.27*E*‐07	-2.04	1.60*E*‐06
CARMN	ENSG00000249669	-6.18	4.66*E*‐15	-4.67	2.23*E*‐11	-3.02	7.51*E*‐09	-4.10	1.78*E*‐11
FGF14-AS2	ENSG00000272143	-2.23	2.36*E*‐06	-2.90	1.26*E*‐13	-2.41	2.13*E*‐09	-2.57	9.86*E*‐10
FZD10-AS1	ENSG00000250208	-2.62	7.84*E*‐14	-2.66	3.70*E*‐14	-2.08	7.17*E*‐09	-2.57	7.81*E*‐12
GAS1RR	ENSG00000226237	-2.38	6.27*E*‐09	-2.68	3.76*E*‐10	-2.18	1.95*E*‐08	-2.43	6.91*E*‐09
GLIDR	ENSG00000278175	-2.89	5.85*E*‐11	-3.65	1.61*E*‐11	-2.02	3.05*E*‐07	-2.99	1.61*E*‐11
HOTAIRM1	ENSG00000233429	-3.59	4.33*E*‐08	-3.80	9.15*E*‐09	-3.39	2.07*E*‐09	-3.89	8.96*E*‐12
HSD11B1-AS1	ENSG00000227591	-5.50	3.99*E*‐13	-5.87	1.55*E*‐10	-5.65	6.09*E*‐10	-5.94	1.42*E*‐10
LINC00358	ENSG00000229578	-2.25	7.11*E*‐11	-2.27	1.52*E*‐08	-2.19	6.90*E*‐08	-2.21	6.47*E*‐08
LINC00408	ENSG00000226250	-6.38	1.23*E*‐22	-6.15	2.14*E*‐18	-4.85	9.77*E*‐14	-5.02	6.61*E*‐15
LINC00968	ENSG00000246430	-2.17	1.36*E*‐06	-2.20	1.63*E*‐05	-2.18	2.13*E*‐05	-2.21	1.29*E*‐05
LINC01140	ENSG00000267272	-3.15	2.57*E*‐07	-3.20	3.98*E*‐06	-2.15	1.27*E*‐03	-2.65	8.56*E*‐06
LINC01192	ENSG00000241369	-2.38	2.37*E*‐08	-2.42	6.29*E*‐07	-2.51	4.75*E*‐07	-2.57	2.07*E*‐07
LINC01697	ENSG00000232079	-5.65	7.26*E*‐18	-5.65	7.39*E*‐15	-4.53	1.53*E*‐09	-5.73	7.03*E*‐15
LINC01720	ENSG00000231175	-2.05	1.58*E*‐11	-2.06	1.88*E*‐09	-2.02	3.89*E*‐09	-2.03	1.08*E*‐09
LINC02053	ENSG00000241696	-5.70	6.69*E*‐18	-5.53	1.07*E*‐13	-5.59	6.05*E*‐13	-5.07	2.61*E*‐12
LINC02535	ENSG00000234155	-2.09	8.56*E*‐11	-2.13	5.70*E*‐09	-2.15	6.59*E*‐09	-2.12	1.07*E*‐08
MEG3	ENSG00000214548	-6.39	3.25*E*‐15	-3.67	3.67*E*‐06	-2.37	9.12*E*‐05	-3.64	1.20*E*‐07
MIR497HG	ENSG00000267532	-2.69	2.77*E*‐17	-2.82	1.69*E*‐14	-2.34	1.84*E*‐11	-2.68	3.47*E*‐13
PGM5-AS1	ENSG00000224958	-2.35	2.44*E*‐06	-3.22	4.17*E*‐09	-2.18	5.62*E*‐07	-2.35	4.72*E*‐06
PRICKLE2-AS3	ENSG00000226017	-2.26	2.07*E*‐09	-2.13	6.53*E*‐07	-2.15	1.11*E*‐06	-2.31	9.74*E*‐08
SNHG29	ENSG00000175061	-3.47	5.31*E*‐10	-4.40	5.21*E*‐09	-2.37	2.28*E*‐07	-3.57	5.97*E*‐09
TRHDE-AS1	ENSG00000236333	-2.68	3.80*E*‐08	-2.87	1.82*E*‐08	-2.63	1.08*E*‐07	-2.68	3.80*E*‐08
WARS2-AS1	ENSG00000231365	-2.21	1.39*E*‐11	-2.49	1.46*E*‐12	-2.18	5.92*E*‐09	-2.31	4.35*E*‐09
ZNF436-AS1	ENSG00000249087	-2.88	1.03*E*‐10	-3.10	2.83*E*‐10	-2.57	6.59*E*‐09	-2.75	2.95*E*‐09

The five DElncRNAs upregulated in all the four breast cancer subtypes were marked with “^∗^”.

## Data Availability

The public datasets GSE45827 and GSE58812 were downloaded from the NCBI Gene Expression Omnibus (GEO). The data used to support the finding of this study are available from corresponding author upon request.

## References

[1] Bray F., Ferlay J., Soerjomataram I., Siegel R. L., Torre L. A., Jemal A. (2018). Global cancer statistics 2018: GLOBOCAN estimates of incidence and mortality worldwide for 36 cancers in 185 countries. *CA: A Cancer Journal for Clinicians*.

[2] Sorlie T., Perou C. M., Tibshirani R. (2001). Gene expression patterns of breast carcinomas distinguish tumor subclasses with clinical implications. *Proceedings of the National Academy of Sciences of the United States of America*.

[3] Carey L. A., Dees E. C., Sawyer L. (2007). The triple negative paradox: primary tumor chemosensitivity of breast cancer subtypes. *Clinical Cancer Research*.

[4] Goldhirsch A., Winer E. P., Coates A. S. (2013). Personalizing the treatment of women with early breast cancer: highlights of the St Gallen International Expert Consensus on the Primary Therapy of Early Breast Cancer 2013. *Annals of Oncology*.

[5] Ades F., Zardavas D., Bozovic-Spasojevic I. (2014). Luminal B breast cancer: molecular characterization, clinical management, and future perspectives. *Journal of Clinical Oncology*.

[6] Juríková M., Danihel Ľ., Polák Š., Varga I. (2016). Ki67, PCNA, and MCM proteins: markers of proliferation in the diagnosis of breast cancer. *Acta Histochemica*.

[7] Turashvili G., Brogi E. (2017). Tumor heterogeneity in breast cancer. *Frontiers in Medicine*.

[8] Pan X., Hu X., Zhang Y. H. (2019). Identification of the copy number variant biomarkers for breast cancer subtypes. *Molecular Genetics and Genomics*.

[9] Li G., Hu J., Hu G. (2017). Biomarker studies in early detection and prognosis of breast cancer. *Advances in Experimental Medicine and Biology*.

[10] Hauptman N., Glavač D. (2013). Long non-coding RNA in cancer. *International Journal of Molecular Sciences*.

[11] Mercer T. R., Dinger M. E., Mattick J. S. (2009). Long non-coding RNAs: insights into functions. *Nature Reviews Genetics*.

[12] Kim J., Piao H. L., Kim B. J. (2018). Long noncoding RNA MALAT1 suppresses breast cancer metastasis. *Nature Genetics*.

[13] Sha S., Yuan D., Liu Y., Han B., Zhong N. (2017). Targeting long non-coding RNA DANCR inhibits triple negative breast cancer progression. *Biology Open*.

[14] Jadaliha M., Gholamalamdari O., Tang W. (2018). A natural antisense lncRNA controls breast cancer progression by promoting tumor suppressor gene mRNA stability. *PLoS Genetics*.

[15] Wang S., Ke H., Zhang H. (2018). LncRNA MIR100HG promotes cell proliferation in triple-negative breast cancer through triplex formation with p27 loci. *Cell Death & Disease*.

[16] Pawlowska E., Szczepanska J., Blasiak J. (2017). The long noncoding RNA HOTAIR in breast cancer: does autophagy play a role?. *International Journal of Molecular Sciences*.

[17] Hashim A., Rizzo F., Marchese G. (2014). RNA sequencing identifies specific PIWI-interacting small non-coding RNA expression patterns in breast cancer. *Oncotarget*.

[18] Zhang W., Shi S., Jiang J., Li X., Lu H., Ren F. (2017). LncRNA MEG3 inhibits cell epithelial-mesenchymal transition by sponging miR-421 targeting E-cadherin in breast cancer. *Biomedicine & Pharmacotherapy*.

[19] Müller V., Oliveira-Ferrer L., Steinbach B., Pantel K., Schwarzenbach H. (2019). Interplay of lncRNA H19/miR-675 and lncRNA NEAT1/miR-204 in breast cancer. *Molecular Oncology*.

[20] Koduru S. V., Tiwari A. K., Leberfinger A. (2017). A comprehensive NGS data analysis of differentially regulated miRNAs, piRNAs, lncRNAs and sn/snoRNAs in triple negative breast cancer. *Journal of Cancer*.

[21] Lehmann B. D., Bauer J. A., Chen X. (2011). Identification of human triple-negative breast cancer subtypes and preclinical models for selection of targeted therapies. *The Journal of Clinical Investigation*.

[22] Zhang Y., Wagner E. K., Guo X. (2016). Long intergenic non-coding RNA expression signature in human breast cancer. *Scientific Reports*.

[23] Wang C. H., Shi H. H., Chen L. H., Li X. L., Cao G. L., Hu X. F. (2019). Identification of key lncRNAs associated with atherosclerosis progression based on public datasets. *Frontiers in Genetics*.

[24] Xia W. X., Yu Q., Li G. H. (2019). Identification of four hub genes associated with adrenocortical carcinoma progression by WGCNA. *PeerJ*.

[25] Zhu Z., Jin Z., Deng Y. (2019). Co-expression network analysis identifies four hub genes associated with prognosis in soft tissue sarcoma. *Frontiers in Genetics*.

[26] Yin W., Tang G., Zhou Q. (2019). Expression profile analysis identifies a novel five-gene signature to improve prognosis prediction of glioblastoma. *Frontiers in Genetics*.

[27] Gruosso T., Mieulet V., Cardon M. (2016). Chronic oxidative stress promotes H2AX protein degradation and enhances chemosensitivity in breast cancer patients. *EMBO Molecular Medicine*.

[28] Jézéquel P., Loussouarn D., Guérin-Charbonnel C. (2015). Gene-expression molecular subtyping of triple-negative breast cancer tumours: importance of immune response. *Breast Cancer Research*.

[29] Gautier L., Cope L., Bolstad B. M., Irizarry R. A. (2004). Affy—analysis of affymetrix GeneChip data at the probe level. *Bioinformatics*.

[30] Ritchie M. E., Phipson B., Wu D. (2015). Limma powers differential expression analyses for RNA-sequencing and microarray studies. *Nucleic Acids Research*.

[31] Zhang X., Sun S., Pu J. K. S. (2012). Long non-coding RNA expression profiles predict clinical phenotypes in glioma. *Neurobiology of Disease*.

[32] Langfelder P., Horvath S. (2008). WGCNA: an R package for weighted correlation network analysis. *BMC Bioinformatics*.

[33] Zhang B., Horvath S. (2005). A general framework for weighted gene co-expression network analysis. *Statistical Applications in Genetics and Molecular Biology*.

[34] Shannon P., Markiel A., Ozier O. (2003). Cytoscape: a software environment for integrated models of biomolecular interaction networks. *Genome Research*.

[35] Huang D. W., Sherman B. T., Lempicki R. A. (2009). Systematic and integrative analysis of large gene lists using DAVID bioinformatics resources. *Nature Protocols*.

[36] Tang Y., Horikoshi M., Li W. (2016). ggfortify: unified interface to visualize statistical results of popular R packages. *The R Journal*.

[37] Kassambara A., Kosinski M., Biecek P. (2019). *survminer: drawing survival curves using 'ggplot 2'. R package version 0.4.6*.

[38] Liu L. C., Wang Y. L., Lin P. L. (2019). Long noncoding RNA HOTAIR promotes invasion of breast cancer cells through chondroitin sulfotransferase CHST15. *International Journal of Cancer*.

[39] Vendrell J. A., Magnino F., Danis E. (2004). Estrogen regulation in human breast cancer cells of new downstream gene targets involved in estrogen metabolism, cell proliferation and cell transformation. *Journal of Molecular Endocrinology*.

[40] Gu J. X., Zhang X., Miao R. C. (2019). Six-long non-coding RNA signature predicts recurrence-free survival in hepatocellular carcinoma. *World Journal of Gastroenterology*.

[41] Liu A. N., Qu H. J., Yu C. Y., Sun P. (2018). Knockdown of LINC01614 inhibits lung adenocarcinoma cell progression by up-regulating miR-217 and down-regulating FOXP1. *Journal of Cellular and Molecular Medicine*.

[42] Binabaj M. M., Bahrami A., Bahreyni A. (2018). The prognostic value of long noncoding RNA MEG3 expression in the survival of patients with cancer: a meta-analysis. *Journal of Cellular Biochemistry*.

[43] Kalimutho M., Sinha D., Jeffery J. (2018). CEP55 is a determinant of cell fate during perturbed mitosis in breast cancer. *EMBO Molecular Medicine*.

[44] Zhan Y., Liang X., Li L. (2016). MicroRNA-548j functions as a metastasis promoter in human breast cancer by targeting Tensin1. *Molecular Oncology*.

[45] Zhang C. Y., Yu M. S., Li X., Zhang Z., Han C. R., Yan B. (2017). Overexpression of long non-coding RNA MEG3 suppresses breast cancer cell proliferation, invasion, and angiogenesis through AKT pathway. *Tumour Biology*.

[46] Tang T., Cheng Y., She Q. (2018). Long non-coding RNA TUG1 sponges miR-197 to enhance cisplatin sensitivity in triple negative breast cancer. *Biomedicine & Pharmacotherapy*.

[47] Andres S. A., Bickett K. E., Alatoum M. A., Kalbfleisch T. S., Brock G. N., Wittliff J. L. (2015). Interaction between smoking history and gene expression levels impacts survival of breast cancer patients. *Breast Cancer Research and Treatment*.

[48] Jovov B., Araujo-Perez F., Sigel C. S. (2012). Differential gene expression between African American and European American colorectal cancer patients. *PLoS One*.

[49] Goto K., Oue N., Hayashi T. (2014). Oligophrenin-1 is associated with cell adhesion and migration in prostate cancer. *Pathobiology*.

[50] Zhao Y., Guo S., Sun J. (2012). Methylcap-seq reveals novel DNA methylation markers for the diagnosis and recurrence prediction of bladder cancer in a Chinese population. *PLoS One*.

[51] Kim J. M., Kang H. J., Kim S. Y. (2015). BDNF promoter methylation associated with suicidal ideation in patients with breast cancer. *International Journal of Psychiatry in Medicine*.

[52] Tajbakhsh A., Mokhtari-Zaer A., Rezaee M. (2017). Therapeutic potentials of BDNF/TrkB in breast cancer; current status and perspectives. *Journal of Cellular Biochemistry*.

[53] Angelucci C., D’Alessio A., Iacopino F. (2018). Pivotal role of human stearoyl-CoA desaturases (SCD1 and 5) in breast cancer progression: oleic acid-based effect of SCD1 on cell migration and a novel pro-cell survival role for SCD5. *Oncotarget*.

[54] Tsai H. Y., Fu S. L., Tseng L. M., Chiu J. H., Lin C. H. (2019). hnRNPK S379 phosphorylation participates in migration regulation of triple negative MDA-MB-231 cells. *Scientific Reports*.

[55] Li Y., Zou L., Li Q. (2010). Amplification of LAPTM4B and YWHAZ contributes to chemotherapy resistance and recurrence of breast cancer. *Nature Medicine*.

[56] Zhou X., Li X., Cheng Y. (2014). BCLAF1 and its splicing regulator SRSF10 regulate the tumorigenic potential of colon cancer cells. *Nature Communications*.

[57] Liu F., Dai M., Xu Q. (2018). SRSF10-mediated IL1RAP alternative splicing regulates cervical cancer oncogenesis via mIL1RAP-NF-*κ*B-CD47 axis. *Oncogene*.

[58] Dent R., Trudeau M., Pritchard K. I. (2007). Triple-negative breast cancer: clinical features and patterns of recurrence. *Clinical Cancer Research*.

[59] Pitner M. K., Taliaferro J. M., Dalby K. N., Bartholomeusz C. (2017). MELK: a potential novel therapeutic target for TNBC and other aggressive malignancies. *Expert Opinion on Therapeutic Targets*.

[60] Zhao Y., du T., du L. (2019). Long noncoding RNA LINC02418 regulates MELK expression by acting as a ceRNA and may serve as a diagnostic marker for colorectal cancer. *Cell Death & Disease*.

[61] Yap K. L., Li S., Muñoz-Cabello A. M. (2010). Molecular Interplay of the Noncoding RNA *ANRIL* and Methylated Histone H3 Lysine 27 by Polycomb CBX7 in Transcriptional Silencing of *INK4a*. *Molecular Cell*.

[62] Wu X. S., Wang F., Li H. F. (2017). LncRNA‐PAGBCacts as a microRNAsponge and promotes gallbladder tumorigenesis. *EMBO Reports*.

[63] Niknafs Y. S., Han S., Ma T. (2016). The lncRNA landscape of breast cancer reveals a role for DSCAM-AS1 in breast cancer progression. *Nature Communications*.

[64] Gupta R. A., Shah N., Wang K. C. (2010). Long non-coding RNA HOTAIR reprograms chromatin state to promote cancer metastasis. *Nature*.

[65] Sun H., Wang G., Peng Y. (2015). H19 lncRNA mediates 17*β*-estradiol-induced cell proliferation in MCF-7 breast cancer cells. *Oncology Reports*.

[66] Jiang Y. Z., Liu Y. R., Xu X. E. (2016). Transcriptome analysis of triple-negative breast cancer reveals an integrated mRNA-lncRNA signature with predictive and prognostic value. *Cancer Research*.

